# Smartphone Ownership Among US Adult Cigarette Smokers: 2014 Health Information National Trends Survey (HINTS) Data

**DOI:** 10.2196/jmir.7953

**Published:** 2017-08-31

**Authors:** Jaimee L Heffner, Kristin E Mull

**Affiliations:** ^1^ Fred Hutchinson Cancer Research Center Division of Public Health Sciences Seattle, WA United States

**Keywords:** mHealth, mobile health, tobacco, smoking, nicotine use disorder

## Abstract

**Background:**

Despite increasing interest in smartphone apps as a platform for delivery of tobacco cessation interventions, no previous studies have evaluated the prevalence and characteristics of smokers who can access smartphone-delivered interventions.

**Objective:**

To guide treatment development in this new platform and to evaluate disparities in access to smartphone-delivered interventions, we examined associations of smartphone ownership with demographics, tobacco use and thoughts about quitting, other health behaviors, physical and mental health, health care access, and Internet and technology utilization using a nationally representative sample of US adult smokers.

**Methods:**

Data were from the National Cancer Institute’s 2014 Health Information National Trends Survey 4 (HINTS 4), Cycle 4. This mailed survey targeted noninstitutionalized individuals aged 18 years or older using two-stage stratified random sampling. For this analysis, we restricted the sample to current smokers with complete data on smartphone ownership (n=479).

**Results:**

Nearly two-thirds (weighted percent=63.8%, 248/479) of smokers reported owning a smartphone. Those who were younger (*P*<.001), employed (*P*=.002), never married (*P*=.002), and had higher education (*P*=.002) and income (*P*<.001) had the highest rates of ownership. Smartphone owners did not differ from nonowners on frequency of smoking, recent quit attempts, or future plans to quit smoking, although they reported greater belief in the benefits of quitting (*P*=.04). Despite being equally likely to be overweight or obese, smartphone owners reported greater fruit and vegetable consumption (*P*=.03) and were more likely to report past-year efforts to increase exercise (*P*=.001) and to lose weight (*P*=.02). No differences in health care access and utilization were found. Smartphone owners reported better physical and mental health in several domains and higher access to and utilization of technology and the Internet, including for health reasons.

**Conclusions:**

Smartphone ownership among smokers mirrors many trends in the general population, including the overall rate of ownership and the association with younger age and higher socioeconomic status. Apps for smoking cessation could potentially capitalize on smartphone owners’ efforts at multiple health behavior changes and interest in communicating with health care providers via technology. These data also highlight the importance of accessible treatment options for smokers without smartphones in order to reach smokers with the highest physical and mental health burden and prevent worsening of tobacco-related health disparities as interventions move to digital platforms.

## Introduction

Smartphones are a promising technology platform for the delivery of smoking cessation interventions. Hundreds of cessation apps are currently available, although only a handful have undergone empirical evaluation of efficacy [1-5] and the vast majority have been found lacking in terms of their inclusion of theoretically backed, evidence-based content [6-8]. A major foundational gap in the literature on smartphone apps for smoking cessation is that no previous studies have evaluated, using a nationally representative sample, what proportion of current smokers in the United States own smartphones, or the characteristics of smokers who currently have access to smartphone-delivered interventions versus those who do not. This gap in the literature hinders treatment development in this new platform as well as understanding of disparities in access to smartphone-delivered interventions.

The application of smartphone technologies to tobacco cessation is new; the technology itself is not. The first smartphones were produced in the 1990s, although the mass adoption of smartphones did not occur in the United States until the late 2000s with the release of Apple’s first-generation iPhone. In the decade that followed, ownership of smartphones increased sharply over time—it jumped from 35% in 2011 to 64% in 2014 and 77% in 2016 [9]. After this extremely rapid period of growth, smartphones are now approaching market saturation in the United States and other developed markets [10]

As with any technology, adoption within some segments of the population is lagging due to factors ranging from lack of interest in learning to use a new technology to low affordability. It is not clear whether US population trends in adoption of smartphones generalize to the population of smokers because smoking behavior is much more concentrated among individuals with lower education and income [11]. The “digital divide” between those smokers who currently own smartphones versus those who do not provides useful information about the potential reach of smartphone-delivered cessation interventions and informs how they are designed and marketed.

Population-level data for the United States suggest that smartphone ownership is associated with several demographic characteristics, including younger age and higher educational attainment and income [12]. However, demographics provide an incomplete picture of the differences between smokers who own smartphones and those who do not. Understanding at a population level how these two groups differ on health care access, health behaviors, and status, as well as usage of other technologies beyond the smartphone, offers an opportunity to target smartphone-delivered interventions based on users’ characteristics and needs, and to identify the needs of smokers who require cessation support through modalities other than the smartphone. Regarding user characteristics and needs, population-level data on smokers who own smartphones could elucidate (1) the prevalence of known barriers to quitting (eg, low motivation, mental health symptoms), (2) the proportion of smartphone-owning smokers whose weight or diet and exercise behaviors place them at even greater risk for poor health outcomes, (3) extent of interest in making changes to these other health behaviors, and (4) potential feasibility of smartphone-delivered treatments that require involvement of the health care system (eg, advising users to seek prescription medications or to discuss withdrawal symptoms with a physician, direct messaging with health care providers).

To guide treatment development on the smartphone platform and to evaluate disparities in access to smartphone-delivered interventions, the aim of this study was to answer three key questions using a large, nationally representative sample:

1. What proportion of current smokers own smartphones?
2. Among smokers, what demographic characteristics are associated with smartphone ownership?
3. How do smokers who own smartphones differ from those who do not on tobacco use and thoughts about quitting, other health behaviors, physical and mental health status, health care access and utilization, and technology and Internet usage?

## Methods

### Source of Data

Data used in these analyses were from the National Cancer Institute’s Health Information National Trends Survey 4 (HINTS 4), Cycle 4. This mailed survey was conducted between August and November 2014. It targeted noninstitutionalized individuals living in the United States aged 18 years or older using two-stage stratified random sampling. The first stage involved selection of a stratified sample of households from a marketing list of nonvacant mailing addresses, and the second stage involved sampling of one adult from each sampled household using the next birthday method. Stratification was designed to overenroll Hispanic and African American participants to increase the precision of estimates for these minority groups. Both English and Spanish versions of the survey were used.

Survey mailings followed a modified Dillman procedure [13]. There were four possible mailings. All selected households received the first survey along with a US $2 incentive, followed by a reminder postcard. Nonrespondents received up to two additional survey mailings. Households flagged as being potentially Spanish speaking received both an English- and Spanish-language version of the survey. The overall weighted response rate was 34.44% (3677/13,996 complete, returned surveys). Additional details regarding the sampling methods and survey procedures can be found in the HINTS 4 database user documentation [14].

### Measures

#### Definitions of Current Smoking and Smartphone Ownership

Participants were classified as current smokers if they reported smoking 100 or more cigarettes in their lifetime and, in response to the item “How often do you now smoke cigarettes?” responded “every day” or “some days.” Smartphone ownership was assessed with the item, “Please indicate if you have a smartphone, such as an iPhone, Android, Blackberry, or Windows phone.” Of the 3677 respondents, 498 (15.00%) were classified as current smokers, and 479 (97.4%) of these provided information on smartphone ownership.

#### Demographics

Demographic survey items included age, gender, current occupational status, household income, marital status, highest grade or level of schooling completed, race, and ethnicity. The US Department of Agriculture’s 2013 Rural-Urban Continuum Code was used to classify participants as residing either in metropolitan (codes 1-3) or nonmetropolitan (codes 4-9) areas.

#### Tobacco Use and Thoughts About Quitting

Respondents were asked whether they stopped smoking for one day or longer in the past year because they were trying to quit and whether they were seriously considering quitting in the next 6 months. They were also asked how much they thought quitting smoking would help reduce the harmful effects of smoking, with response options ranging from “not at all” to “a lot.”

#### Other Health Behaviors

Assessment of current health behaviors included minutes per week of at least moderate exercise (the product of two questions: “In a typical week, how many days do you do any physical activity or exercise of at least moderate intensity, such as brisk walking, bicycling at a regular pace, and swimming at a regular pace” and “On the days that you do any physical activity or exercise of at least moderate intensity, how long do you typically do these activities?”), which was transformed to a binary categorical variable indicating whether or not the respondent met the recommended minimum weekly moderate-intensity exercise duration of 150 minutes [15]. Fruit and vegetable consumption, in total cups per day (“About how many cups of fruit [including 100% pure fruit juice] do you eat or drink each day?” and “About how many cups of vegetables [including 100% pure vegetable juice] do you eat or drink each day?”), were assessed separately as a range, transformed to a continuous variable by taking the midpoint of the range, and combined for analysis. Several items assessed health behavior change efforts in the prior year (ie, “At any time in the past year, have you *intentionally* tried to...”), including attempts to change physical activity and fruit and vegetable consumption and to lose weight.

#### Physical and Mental Health Status

Body mass index (BMI) was calculated from the respondent’s self-reported height and weight and used to classify participants as overweight or obese (BMI ≥25.0 kg/m^2^). Physical health status was assessed, in part, by asking whether the respondent had ever been diagnosed with diabetes, hypertension, a heart condition, lung disease, arthritis, and cancer. In terms of mental health, the four-item Patient Health Questionnaire (PHQ-4) [16] was included as a measure of psychological distress. We separated this measure into its component screening tools for depression (PHQ-2) [17] and anxiety (Generalized Anxiety Disorder scale [GAD]-2) [18]. Respondents were also asked whether they had ever been diagnosed with a depressive or anxiety disorder.

#### Health Care Access and Utilization

Respondents indicated whether or not they had health care coverage as well as how many times they received nonemergent medical care over the past year.

#### Use of Technology and the Internet

Items included whether or not the respondent used the Internet, device ownership, use of health apps, and which technology-mediated methods they have used to exchange medical information with a health care provider.

### Statistical Analyses

Statistical analyses incorporated weights for survey respondents in order to adjust for nonresponse and noncoverage biases, thus ensuring valid inference. Sample weights and replicate weights were calculated as described in the HINTS 4 Cycle 4 Methodology Report [14]. Briefly, full-sample weights were derived by first adjusting for household-level base weights (ie, the reciprocal of the probability of selecting the household for the survey) and household nonresponse, calculating initial weights, and finally calibrating these to population counts. Replicate weights were calculated using the delete one jackknife procedure. We estimated population-level proportions of smartphone ownership and differences between smartphone owners and nonowners on demographics, tobacco use and thoughts about quitting, other health behavior, physical and mental health status, health care access, and use of technology using full-sample weights. Thus, the weighted percentages do not directly correspond with the raw sample proportions. For group comparisons, we implemented chi-square tests of independence and *t* tests using replicate weights. Because a main aim of the study was to better understand characteristics of smokers who own smartphones for intervention planning purposes rather than to identify the set of variables that most efficiently distinguish smartphone-owning smokers from nonowners, we decided against a multivariable approach. However, we accounted for multiple comparisons by adjusting *P* values to control the false discovery rate (FDR) [19].

## Results

### Proportion of Current Smokers in the United States Who Own Smartphones

Overall, the weighted proportion of current smokers who owned smartphones was 63.8% (248/479). Of those who did not own smartphones (231/479), 53.6% (128/231) owned a basic cell phone. Among smartphone owners, 36.7% (88/245) reported using a health app on their phone or tablet.

### Demographic Characteristics of Smokers Associated with Smartphone Ownership

As shown in Table 1, smartphone ownership among smokers differed according to age, education, income, employment, and marital status. There was decreasing prevalence of smartphone ownership from the 18 to 34 years age group (88%, 59/68) through the 65 years and older age group (15%, 17/85; *P*<.001). Increasing levels of education (*P*=.002) and income (*P*<.001) also showed nearly linear increases in smartphone ownership. More than 84% (76/112) of respondents with a college degree or higher and more than 90% (63/80) of those with household incomes of at least US $75,000 per year owned smartphones. In terms of employment status, employed respondents had the highest rates of ownership (74.1%, 155/224; *P*=.002). Those who were divorced, widowed, or separated (36.3%, 75/171) had lower rates of ownership than those who were married (59.0%, 90/165) or never married (77.3%, 71/122; *P*=.002). Smartphone ownership did not differ by gender, race/ethnicity, or residence in a metropolitan versus nonmetropolitan area.

**Table 1:** Prevalence of smartphone ownership among smokers by demographic characteristics (n=479).

Demographic characteristic		Prevalence of smartphone ownership, n (%)^a^	*P*^b^
**Age (n=457)**			<.001
	18-34 (n=68)	59 (88)	
	35-49 (n=92)	64 (67)	
	50-64 (n=212)	114 (47.9)	
	≥65 (n=85)	17 (15)	
**Gender (n=448)**			.93
	Female (n=265)	144 (64.6)	
	Male (n=183)	88 (64.0)	
**Education (n=458)**			.002
	Less than high school (n=57)	19 (38)	
	High school graduate (n=116)	46 (55.1)	
	Some college (n=173)	95 (66.8)	
	College grad or more (n=112)	76 (84.1)	
**Race/ethnicity (n=419)**			.93
	Non-Hispanic white (n=255)	138 (66.1)	
	Non-Hispanic black (n=89)	50 (67)	
	Hispanic (n=47)	24 (61)	
	Non-Hispanic other (n=28)	16 (78)	
**Income (US$) (n=475)**			<.001
	0-14,999 (n=140)	44 (45.4)	
	15,000-34,999 (n=124)	60 (52.9)	
	35,000-74,999 (n=131)	80 (64.8)	
	75,000-99,000 (n=34)	28 (93)	
	≥100,000 (n=46)	35 (90)	
**Residence (n=479)**			.07
	Metro (n=409)	216 (68.1)	
	Non-metro (urban or rural) (n=70)	32 (47)	
**Employment (n=445)**			.002
	Employed (n=224)	155 (74.1)	
	Unemployed (n=41)	20 (60)	
	Retired (n=76)	19 (31)	
	Disabled (n=76)	24 (40)	
	Other (student, homemaker, other) (n=28)	16 (50)	
**Marital status (n=458)**			.002
	Married/living as married (n=165)	90 (59.0)	
	Divorced/widowed/separated (n=171)	75 (36.3)	
	Never married (n=122)	71 (77.3)	

^a^Percentage values in table are weighted by the overall sample weight.

^b^*P* values were calculated using chi-square tests for independence on the weighted percentages.

**Table 2:** Differences between smartphone-owning and nonowning smokers on health behaviors, health status, health care access and utilization, and Internet and technology use.

Variable		Smartphone owners^a^ (n=248)	Nonowners^a^ (n=231)	*P*^b^
**Tobacco use behavior and attitudes, n (%)**				
	Daily smoker (n=479)	173 (68.5)	192 (80.3)	.20
	Made quit attempt in past year (n=476)	159 (59.5)	136 (55.6)	.71
	Considering quitting in next 6 months (n=472)	167 (63.4)	136 (57.8)	.51
	Believe that quitting reduces harm of smoking “a lot” (n=476)	189 (83.7)	147 (62.0)	.04
**Other health behaviors**				
	Gets recommended ≥150 min/week of moderate exercise, n (%) (n=474)	115 (54.8)	78 (43.6)	.30
	Total cups of fruit and vegetable consumption per day, mean (SD) (n=470)	2.7 (0.2)	2.0 (0.2)	.03
	Tried to increase exercise in past year, n (%) (n=465)	134 (59.8)	68 (26.2)	.001
	Tried to change weight in past year, n (%) (n=466)	138 (55.5)	83 (31.9)	.02
	Tried to increase fruit or veg consumption in past year, n (%) (n=459)	123 (42.6)	81 (38.9)	.72
**Physical and mental health status, n (%)**				
	Overweight/obese (BMI ≥25.0) (n=463)	149 (63.3)	135 (54.9)	.47
	Positive screen for current depression (n=463)	52 (12.9)	66 (37.5)	.02
	Positive screen for current anxiety (n=460)	51 (17.2)	57 (32.7)	.10
	Ever diagnosed with depression/anxiety disorder (n=466)	77 (22.0)	97 (45.1)	.04
	Ever diagnosed with diabetes (n=466)	32 (7.3)	58 (21.3)	.02
	Ever diagnosed with of hypertension (n=468)	96 (24.6)	117 (39.3)	.02
	Ever diagnosed with heart condition (n=468)	21 (5.1)	32 (9.6)	.30
	Ever diagnosed with lung disease (n=467)	49 (13.4)	56 (22.3)	.23
	Ever diagnosed with arthritis (n=466)	65 (17.8)	93 (34.5)	.04
	Ever diagnosed with cancer (n=477)	25 (4.5)	26 (7.4)	.30
**Health care access and utilization, n (%)**				
	Have health care coverage (n=478)	192 (78.2)	184 (84.6)	.32
	Visited health care provider in past year (excluding ER) (n=469)	187 (69.8)	187 (78.5)	.36
**Technology and Internet usage, n (%)**				
	Use Internet (n=479)	224 (95.1)	127 (52.5)	.001
	Have tablet (n=479)	139 (59.0)	47 (24.6)	.001
	Exchanged medical information by email with a provider (n=474)	69 (24.7)	22 (6.6)	.03
	Exchanged medical information by text with a provider (n=474)	22 (5.7)	15 (4.8)	.72
	Exchanged medical information by app with a provider (n=474)	19 (6.6)	7 (3.9)	.51
	Exchanged medical information by video conference with a provider (n=474)	2 (0.4)	5 (2.1)	.36
	Exchanged medical information by social media with a provider (n=474)	15 (4.4)	8 (4.3)	.98

^a^Percentage values as well as mean and standard deviation in table are weighted by the overall sample weight.

^b^*P* values were calculated using chi-square tests for independence on the weighted percentages.

### Differences by Smartphone Ownership in the Characteristics and Behaviors of Smokers

Comparisons of smokers who own smartphones and those who do not are provided in Table 2.

#### Tobacco Use and Thoughts About Quitting

Smartphone owners did not differ significantly from nonowners on daily versus nondaily smoking, recent quit attempts, or plans to quit in the next 6 months. They did endorse greater belief that quitting smoking reduces smoking-related harm (*P*=.04).

#### Other Health Behaviors

Smartphone owners reported higher levels of fruit and vegetable consumption (2.7 vs 2.0 cups per day, *P*=.03). A majority had, in the past year, tried to increase exercise (59.8%, 134/240) and lose weight (55.5%, 138/241), which was significantly more common than among those smokers who did not own smartphones (26.2%, 68/225 tried to increase exercise and 31.9%, 83/225 tried to lose weight; *P*=.001 and *P*=.02, respectively). The two groups did not differ on adherence to the recommendation of 150 or more minutes per week of moderate exercise or on attempts to increase fruit and vegetable consumption in the past year.

#### Physical and Mental Health Status

Despite the aforementioned finding that smartphone owners reported higher levels of fruit and vegetable consumption and were more likely to have engaged in recent efforts to increase exercise and lose weight, smartphone owners were no less likely to be overweight or obese than those smokers who did not own smartphones (63.3%, 149/243 vs 54.9%, 135/220; *P*=.47). Smartphone owners also reported lower rates of some, but not all, physical and mental health problems. They were less likely to screen positive for depression on the PHQ-2 (12.9%, 52/241 vs 37.5%, 66/222; *P*=.02) and less likely to report ever having been diagnosed with a depressive or anxiety disorder (22.0%, 77/242 vs 45.1%, 97/224; *P*=.04), diabetes (7.3%, 32/244 vs 21.3%, 58/222; *P*=.02), hypertension (24.6%, 96/244 vs 39.3%, 117/224; *P*=.02), and arthritis (17.8%, 65/242 vs 34.5%, 93/224; *P*=.04). They did not differ on current anxiety or on lifetime diagnosis of lung disease, heart condition, or cancer.

#### Health Care Access and Utilization

Smokers with smartphones were no more likely to have health care coverage (78.2%, 192/248) than those without smartphones (84.6%, 184/230; *P*=.32). They were also equally likely to have received nonemergency medical care in the prior year (69.8%, 187/244 vs 78.5%, 187/225; *P*=.36).

#### Technology and Internet Usage

Nearly all (95.1%, 224/248) smartphone owners reported using the Internet, whereas closer to half (52.5%, 127/231) of those who did not own smartphones reported Internet use (*P*=.001). Smartphone owners were more than twice as likely to own tablet computers (59.0%, 139/248 vs 24.6%, 47/231; *P*=.001). In terms of technology-mediated communication with health care providers, smartphone owners were more likely to have communicated via email (24.7%, 69/247 vs 6.6%, 22/227; *P*=.03) with their provider. There were no differences in communication with a provider by text, app, video conference, or social media, but overall prevalence of use of these methods of communication was very low in both groups (0%-7%).

## Discussion

This was the first study to use a nationally representative sample to estimate the prevalence of smartphone ownership among current smokers in the United States and to evaluate the relationship between smartphone ownership and demographics, tobacco use and thoughts about quitting, other health behaviors, physical and mental health, health care access, and Internet and technology utilization. Regarding the prevalence of smartphone ownership among smokers, the 63.8% weighted estimate for this late-2014 HINTS 4 sample of adult current smokers is nearly identical to the 64% rate of smartphone ownership for the US population in late 2014 reported by the Pew Research Center [12]. Where smartphone ownership data specific to smokers are not available, this finding suggests that it is reasonable to assume that population estimates of smartphone ownership among smokers follow the broader population trends. If that is the case, current rates of smartphone ownership among smokers should be approximately 77% based on the 2017 Pew survey results [9].

Considering these ownership data, smartphone apps for smoking cessation have high potential reach. Although the HINTS survey does not assess usage of smoking cessation apps specifically, it does assess use of health apps more broadly. We found that over one-third of smartphone-owning smokers (36.7%) had ever used a health app. The latest Pew survey results on mobile health app use in the general population, which were reported in 2012, found that 19% of adults reported having health apps on their phones [20]. A more recent survey showed much higher rates health app use (58%) [21]. These findings point toward an increase in health app usage over time. Only one prior study has specifically examined cessation app use among smokers, finding that 15% of US adult smokers had ever used a cessation app and 43% were interested in using an app in the future [22]. Taken together with our finding that a high proportion of the 42 million US smokers [23] own smartphones, these data on the usage of and interest in health apps more broadly, and cessation apps more specifically, indicate that smartphone apps are a promising new approach to assist millions of smokers by expanding access to smoking cessation interventions.

As of 2014, smartphone ownership in the broader US population was associated with younger age, higher educational attainment, and higher income, but not with race or ethnicity [12]. Data from this study mirror these general population trends in smartphone ownership, where the younger smokers and those of higher socioeconomic status are more likely to have access to the technology. With the notable exception of the lack of differences by race or ethnicity, this pattern of findings on the demographics of smartphone ownership is reminiscent of the “digital divide” that emerged in the early days of the Internet [24], restricting which smokers could access Web-assisted tobacco treatment [25]. There is reason to believe that this digital health divide will recur with each new technological advancement, perpetuating disparities in treatment access and necessitating consideration of how to best reach those who remain on the other side of the divide.

Traditional modalities such as face-to-face counseling and telephone quitline counseling are effective yet underutilized alternatives [26] to smartphone-delivered interventions. Other technology-driven methods could also be employed to reach smokers who do not own smartphones. For example, both text messaging and Web-based interventions are effective for smoking cessation [27,28]. Within the group of smokers who did not own smartphones, 53.6% owned a basic cellular phone and 52.5% reported using the Internet, meaning that at least half could access either a text messaging or Web-based program.

Undoubtedly, the demographics of smartphone ownership will continue to change over time and, even now, a substantial proportion of disadvantaged smokers have access to smartphone-delivered treatments. In this HINTS 4 sample, nearly half (45.4%) of smokers in the lowest category of income (US $0-$14,999) reported owning a smartphone. Given the low cost and high accessibility of apps for smokers who own a smartphone, this method of treatment delivery offers many potential benefits for disadvantaged populations of smokers.

Demography is just one facet of understanding the smartphone divide and its implications for treatment development and accessibility. We also evaluated the possibility that smartphone owners differed from nonowners on tobacco use and thoughts about quitting, other health behaviors, physical and mental health, health care access, and technology utilization. The prevalence of daily tobacco use as well as past and planned efforts to quit were similar among smartphone owners and nonowners, although smokers who did not own smartphones expressed less optimism about the health benefits of quitting compared to smokers who did own smartphones. This may be related to the older age and worse mental and physical health reported by this group, including higher rates of depressive symptoms and diagnoses of depression or anxiety disorders, diabetes, hypertension, and arthritis. The clustering of physical and mental health conditions within the group of smokers without smartphones makes this a more challenging population of smokers to treat. As the smartphone-specific digital divide lessens over time, the answer to the question of how effective smartphone apps are for smokers with physical and mental health conditions will become increasingly important. It is also important that tobacco treatment be readily accessible to smokers with physical and mental health conditions through other means, or tobacco-related health disparities will continue to worsen among these vulnerable populations [29].

Surprisingly, we did not observe a divide in health care access between smartphone owners and nonowners. This finding differs from that of an earlier study investigating the digital divide in Web-based tobacco cessation interventions, where those smokers who did not have Internet access were also less likely to have health care access [25]. With the expanded coverage offered by the Affordable Care Act, a majority of smokers should be able to access one or more effective forms of assistance to quit smoking through their health insurance, and those who do not have health care coverage can still access no-cost assistance through a tobacco quitline or websites such as Smokefree.gov. Smoking cessation apps, if proven effective, could expand the safety net of no- or low-cost standalone cessation assistance for the estimated 22% of smartphone-owning smokers who do not have health insurance.

### Implications for Future Research on Smartphone App Design

The observed characteristics of smokers who own smartphones raise a number of questions about the optimal design of cessation programs delivered on this platform. First, we found that smartphone owners were more health-conscious with respect to their greater consumption of fruits and vegetables as well as their greater efforts to increase exercise and lose weight in the previous year, suggesting potential synergies between motivation to change smoking, exercise, and nutrition. Although attempting to lose weight while quitting smoking is typically discouraged [26], exercise and proper nutrition can assist smokers in minimizing weight gain after quitting and may be of interest for smokers using smartphone apps to quit. The extent to which smokers would engage with and benefit from a multiple health behavior change app, as opposed to one that focused exclusively on smoking cessation, is an important topic for future research.

Second, smartphone technology offers a medium for synchronous or asynchronous communication with health care providers to support cessation efforts. Email was used by almost one-quarter of smartphone owners as a method of communicating with providers. This is a substantial proportion, particularly given that the option of secure messaging with providers is not available universally. On the other hand, very few participants used apps, text messaging, video conferencing, or social media for this type of communication. It cannot be determined from these data how many smokers would use these methods to communicate with providers specifically about smoking cessation if that option were available, but smokers indicate that they like the idea of having some form of support built into smoking cessation apps [30], and email or other messaging components within an app provides smokers with supportive accountability for behavior change [31]. Smokers’ interest in communicating with health care providers through cessation apps should be evaluated further in order to identify the most desirable, effective, and secure methods of communication. Additionally, given the high proportion of smartphone-owning smokers in this study who had health insurance (78%) and who had visited a health care provider in the past year (70%), the extent to which cessation apps could be built for integration into the health care system should be evaluated. Such integration could have a number of benefits, including: (1) provider support for use of the programs, which increases adherence by 10-30% [32]; (2) integration of app data into electronic medical records systems for monitoring of symptoms, patient self-management strategies, and treatment response; and (3) offering apps for behavioral support alongside cessation medications to support quitting, which is consistent with current clinical practice guidelines [26] and has the potential to increase adherence to pharmacotherapy [33].

### Limitations

The primary limitation of this analysis stems from the challenge of keeping pace with the speed of technology advancement in population surveys. The HINTS 4, Cycle 4 data were collected in 2014, and changes in smartphone ownership since that time may affect the demographics of smartphone ownership and comparisons between smartphone owners and nonowners. Additionally, the HINTS survey is conducted by the US National Cancer Institute and focuses on US residents; therefore, the results are generalizable only to smokers in the United States. The prevalence and correlates of smartphone ownership among smokers is likely to vary considerably across countries. As described in the Methods section, we accounted for multiple comparisons by controlling the FDR [19], or the expected proportion of falsely rejected null hypotheses. The FDR is equivalent to the familywise error rate (FWER) when all null hypotheses are true, but it is smaller otherwise. Using the FDR provides a potential gain in power where control of the conservative FWER is unnecessarily stringent [19]. Still, due to the small samples sizes for some comparisons and the *P* value adjustment, results are conservative. Lack of statistical significance should therefore not be interpreted as a conclusive demonstration of no effect and, depending on the context in which they are applied, differences that are not statistically significant may still have practical significance. As such, this research is best characterized as exploratory. Finally, our analyses focus on bivariate correlations between constructs measured at a single time point; thus, we cannot evaluate the effects of time or rule out confounding factors that influence bivariate correlations.

### Conclusions

Smartphone ownership among US smokers mirrors many trends in the general population, including the overall rate of ownership and the association with younger age and higher socioeconomic status. Smokers who own smartphones are also healthier, more health-conscious, and are higher users of technology and the Internet than those who do not own smartphones. Design of smartphone-delivered cessation interventions would benefit from additional research on the implications of these user characteristics and behavior, including smartphone owners’ interest in multiple health behavior change and interest in communicating with health care providers via technology. These data also highlight the importance of continuing to offer a broad range of intervention strategies that do not require smartphones for access in order to reach the smokers with the highest physical and mental health burden and prevent worsening of tobacco-related health disparities.

## Abbreviations

BMI: body mass index
FDR: false discovery rate
FWER: familywise error rate
GAD: generalized anxiety disorder
HINTS: Health Information National Trends Survey
PHQ: Patient Health Questionnaire
